# Supplementary material for: The Redesign of the Molecular Scaffold of Viral Ion Channel Blockers
Source: Comput Struct Biotechnol J. 2026 Aug 3;35(1):0188. doi: 10.34133/csbj.0188 (PMC13430562; doi:10.34133/csbj.0188)
Supplement: Supplementary 1 — Figs. S1 to S7 Tables S1 to S4 [file csbj.0188.f1.docx]

**Supporting Information**

**The redesign of the molecular scaffold of viral ion channel blockers**

Balázs Zoltán Zsidó^1^, Erzsébet Mernyák^2^, Zoltán Kopasz^3,4^, Fanni Földes^3^, Krisztina Leiner^3,4^, Mónika Madai^3^, Brigitta Zana^3^, Anett Kuczmog^3,4^*, Csaba Hetényi^1^*

*^1^Pharmacoinformatics Unit, Department of Pharmacology and Pharmacotherapy, Medical School, University of Pécs, Szigeti út 12, 7624 Pécs, Hungary.*

*^2^Institute of Pharmacognosy, University of Szeged, Eötvös u. 6, H-6720 Szeged, Hungary*

*^3^National Laboratory of Virology, Szentágothai Research Centre, University of Pécs, Ifjúság útja 20, 7624 Pécs, Hungary.*

*^4^Institute of Biology, Faculty of Sciences, University of Pécs, Ifjúság útja 6, 7624 Pécs, Hungary.*

*Corresponding authors.

**Table of contents**

Figure S1 2

Figure S2 3

Figure S3 4

Figure S4 5

Figure S5 6

Figure S6 7

Figure S7 8

Table S1 9

Table S2 10

Table S3 11

Table S4 12

References 13


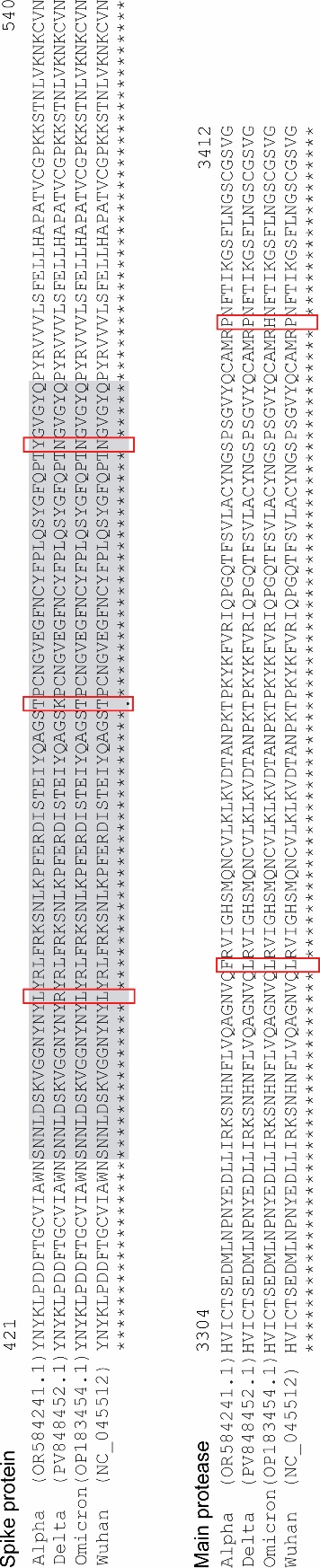


**Figure S1:** The amino acid sequence alignment of the spike protein, and main protease from different SARS-CoV-2 variants studied here. Sequences were obtained from the National Center for Biotechnology Information, with repository codes shown in the figure. Sequence alignment was performed using Clustal Omega [1]. The receptor-binding site of the spike protein is colored gray, following [2].


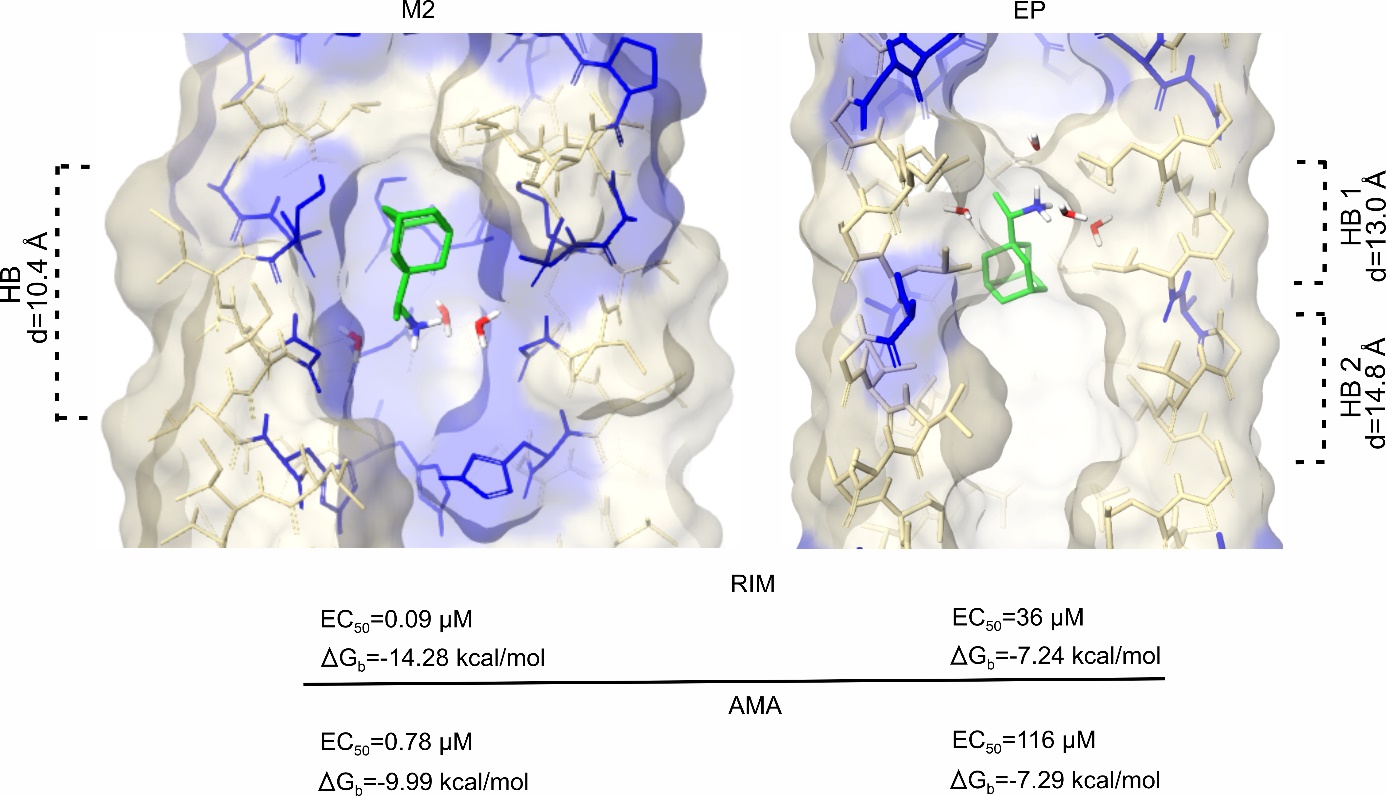


**Figure S2:** The binding of RIM and AMA to the M2 ion channel of influenza A virus and the EP ion channel of SARS-CoV-2 [3]. The spatial binding mode of RIM is shown at the top. RIM is depicted in an all-atom representation, with water molecules as red and white sticks and the ion channels as a transparent surface with sticks. Hydrophobic bands (HB) are displayed and labeled by diameter. The experimental structure of RIM-M2 shows that RIM fits into a narrow hydrophobic band with a diameter (d) of 10.4 Å in M2, located between two hydrophilic bands. The predicted binding mode of RIM in EP does not match the hydrophilic/hydrophobic bands as well as in M2. RIM binds to a larger HB1 in EP (d=13.0 Å) compared to the 10.4 Å in M2, resulting in weaker antiviral activity (Table S2) and binding (Table S1) to EP relative to M2.


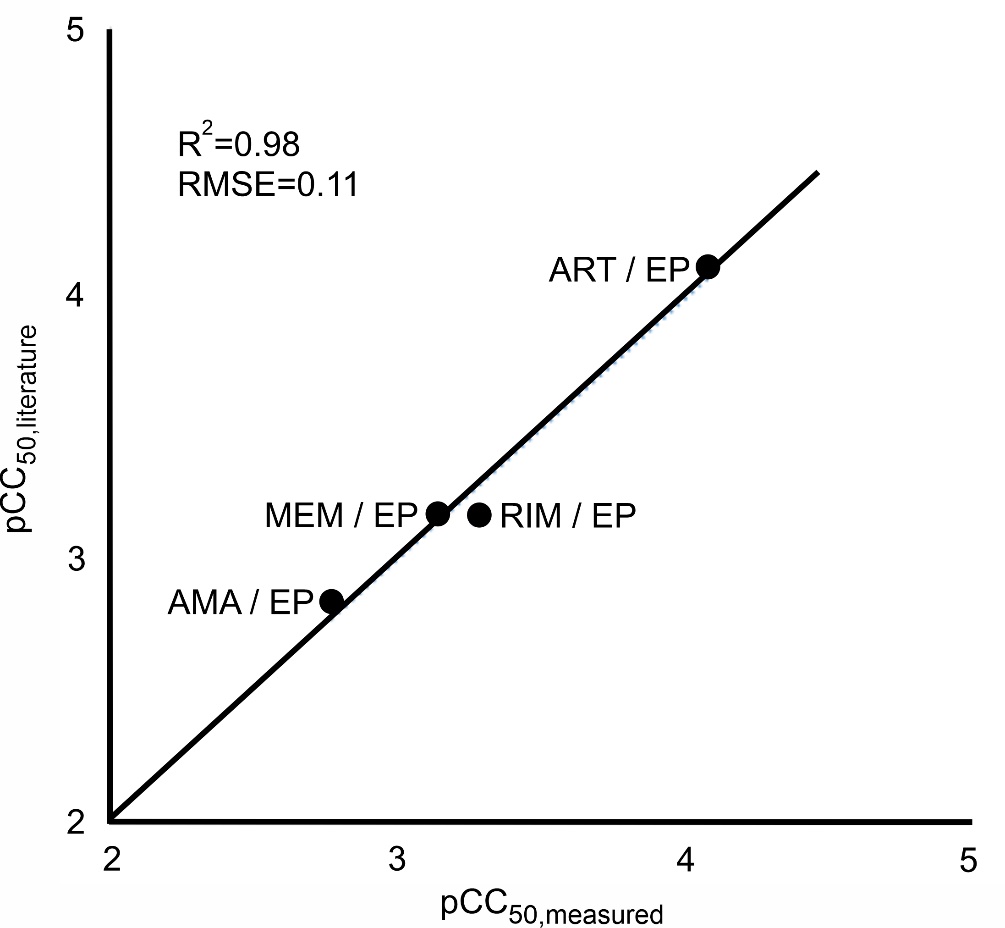


**Figure S3:** Correlation between CC_50_ values measured by other groups (Table S2) and in the present study (Table S3). The points are labeled according to drug/target.


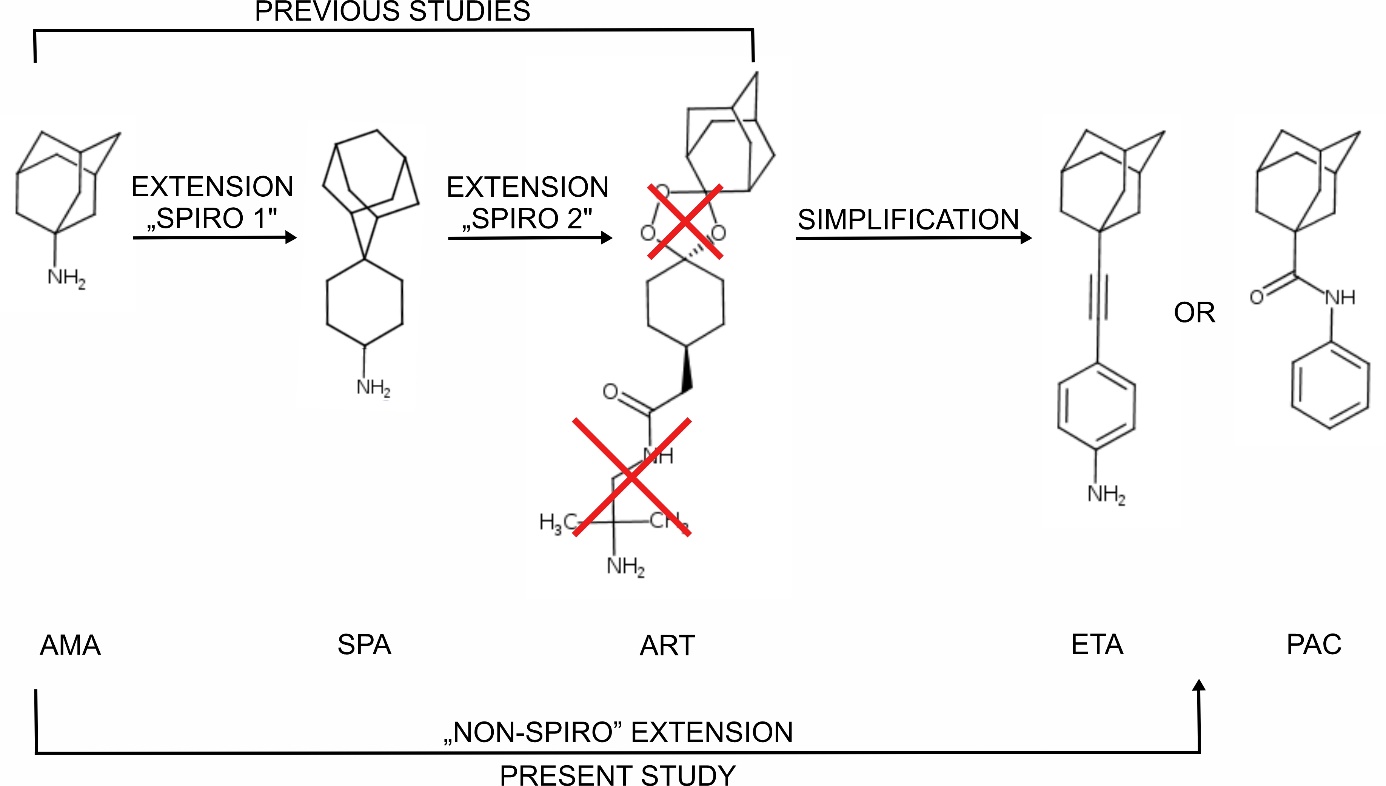


**Figure S4:** Previous studies extended the scaffold of AMA with spiro-compounds SPA and ART, which have certain drawbacks described in the main text. In this study, an additional phenyl ring was attached to the adamantyl ring similar to SPA, but instead of a spiro connection, a small amide and ethynyl linker were used to connect the two rings in PAC and ETA, respectively. These linkers are comparable in length to the 1,2,4-trioxolane ring in ART (unnecessary for EP binding). The new linkers can effectively replace the trioxolane ring (red strike through), resulting in the extended AMA derivatives PAC and ETA. The problematic hydrophilic groups of ART (see main text) were also removed from the new compounds (red strike through). This simplified approach produced compounds that are easier to synthesize, still active, and structurally simpler, namely ETA and PAC.


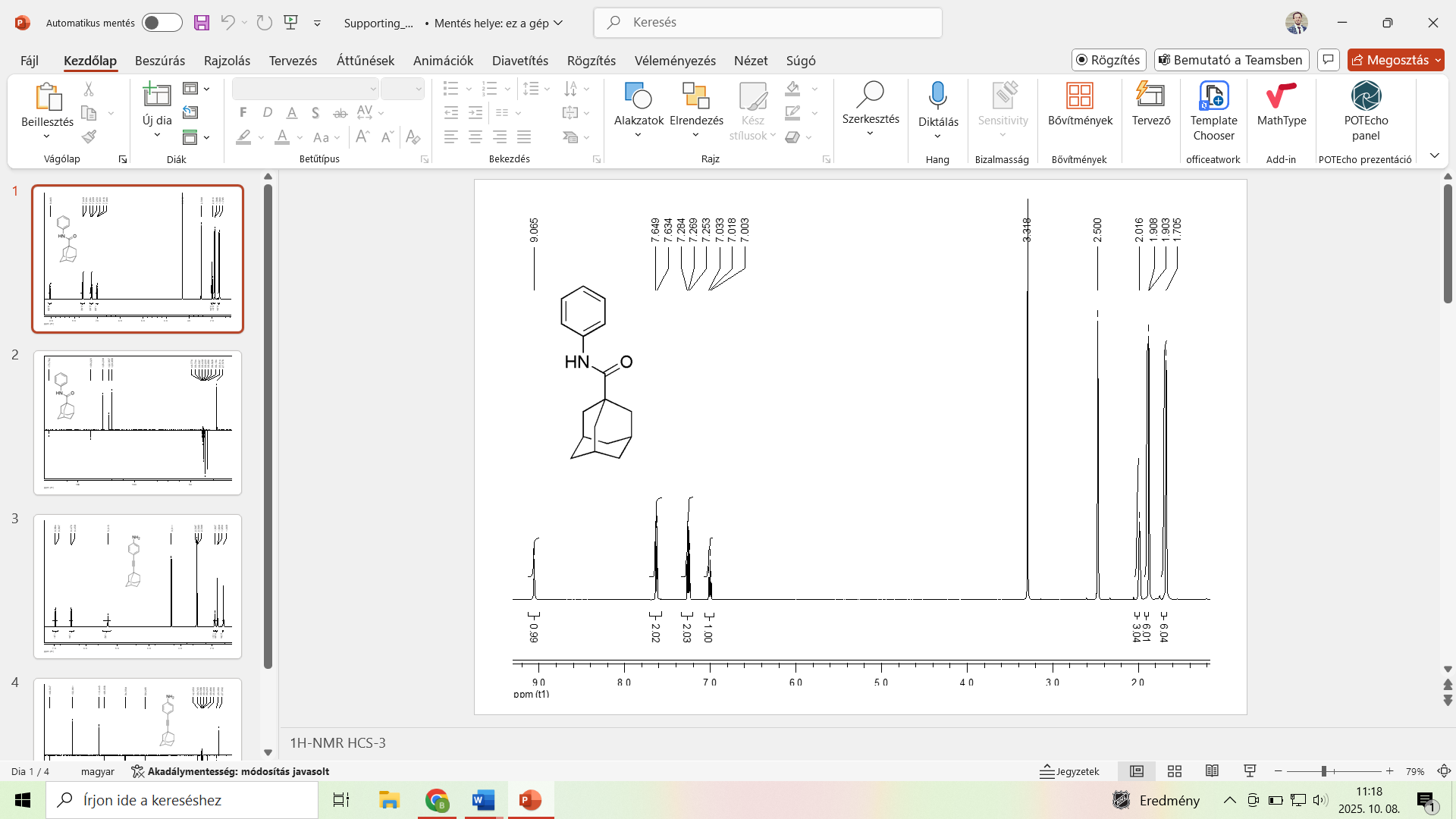


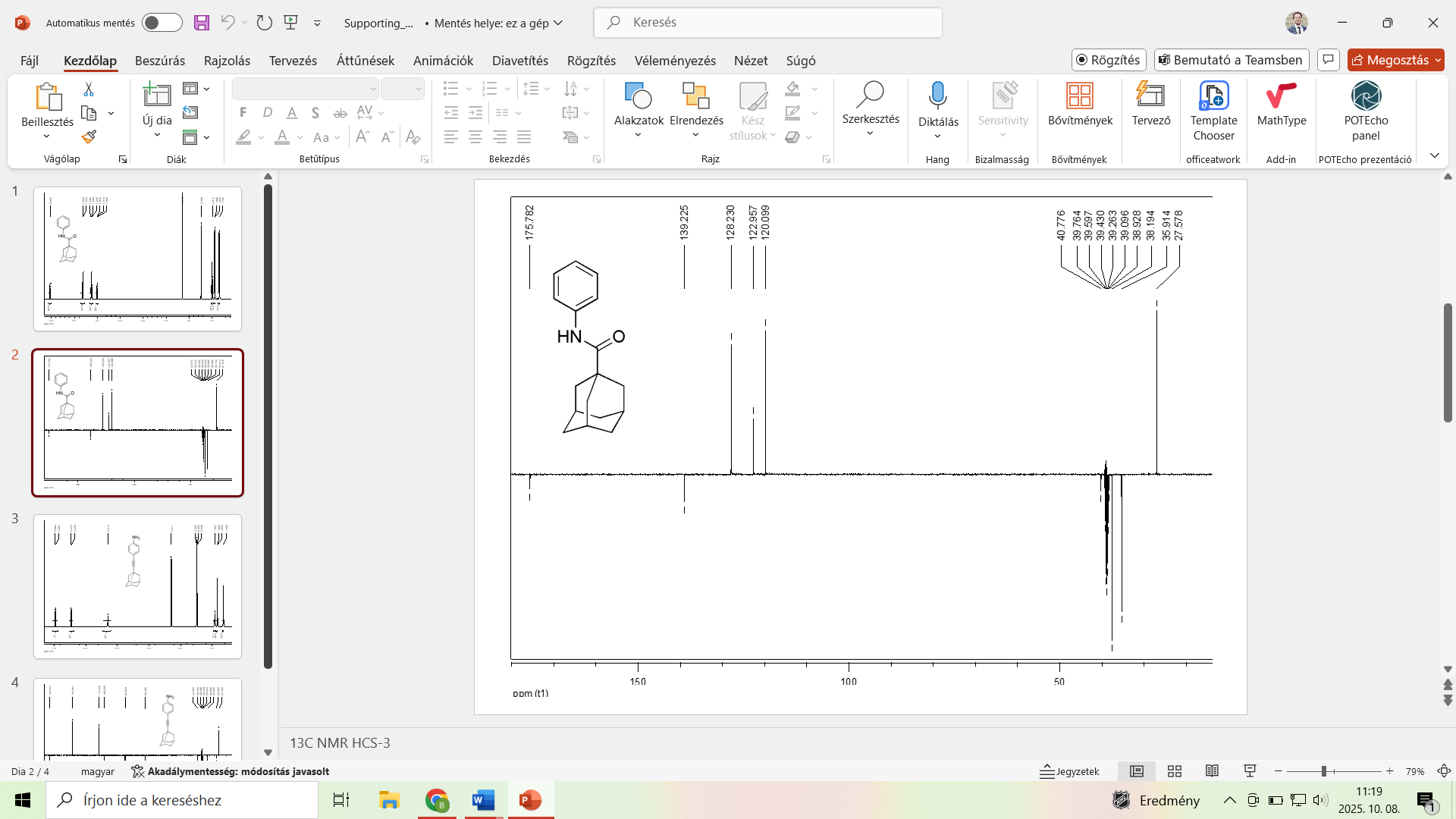


**Figure S5:** ^1^H and ^13^C NMR spectra of PAC


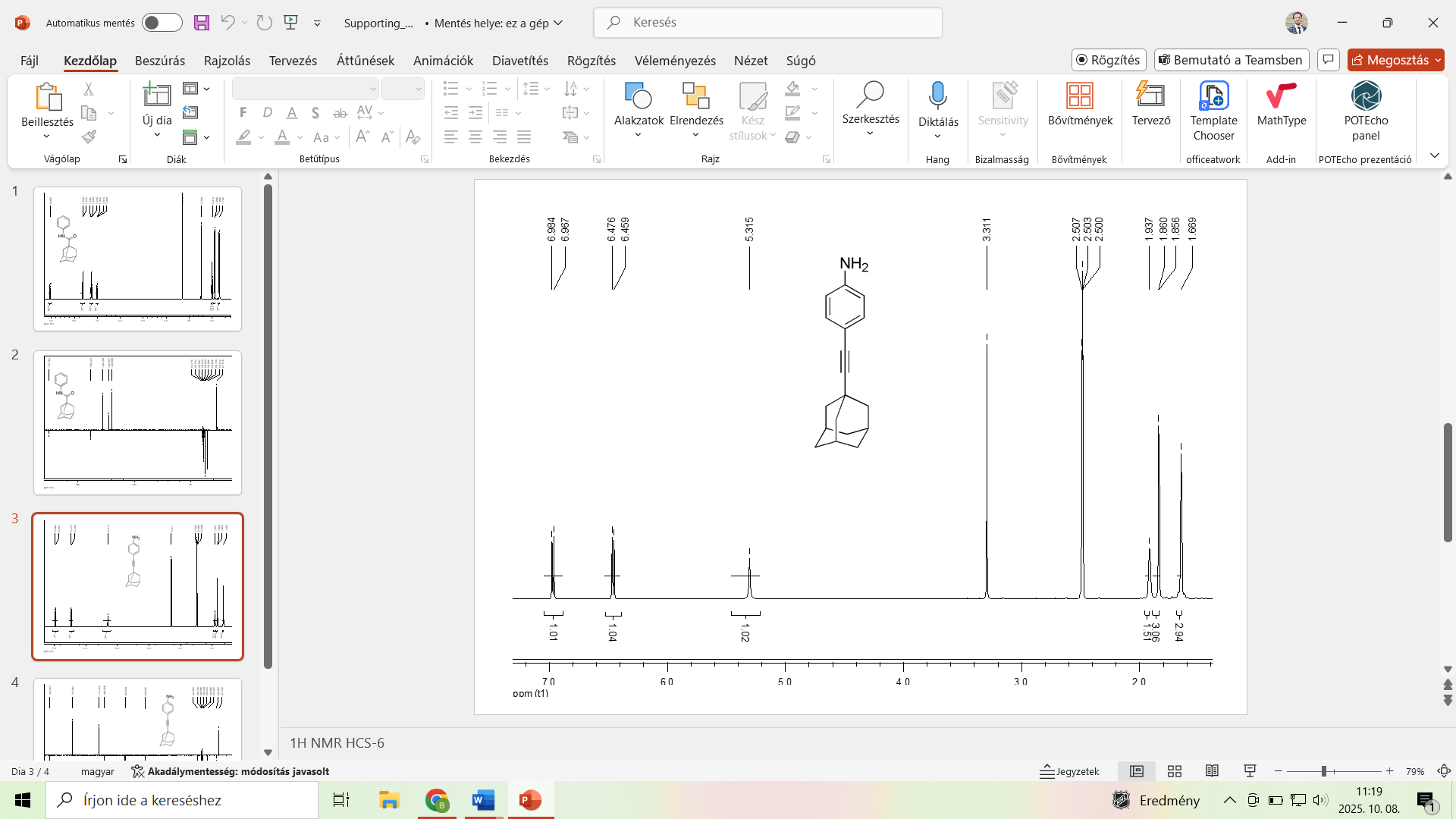


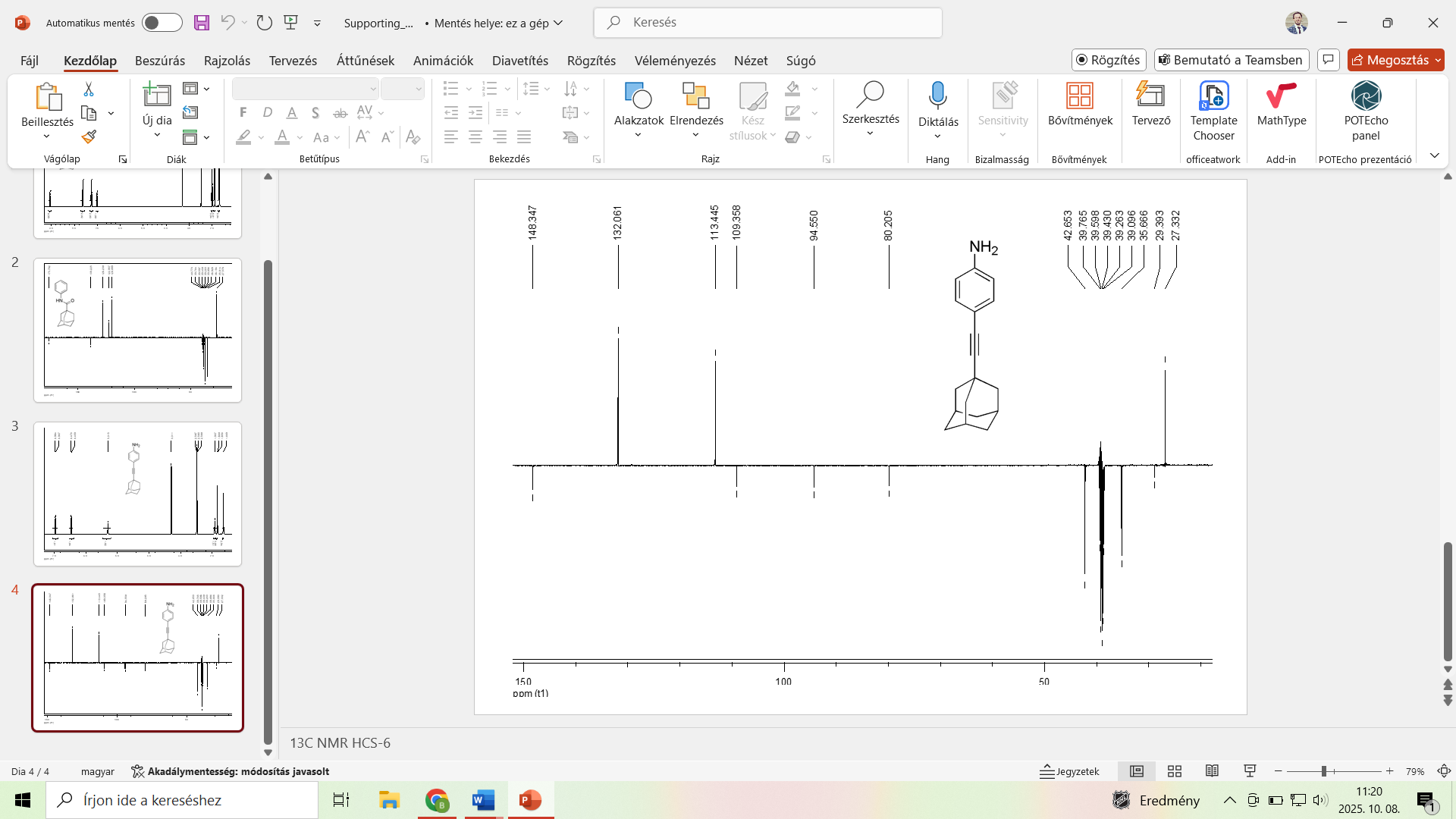


**Figure S6:** ^1^H and ^13^C NMR spectra of ETA

**
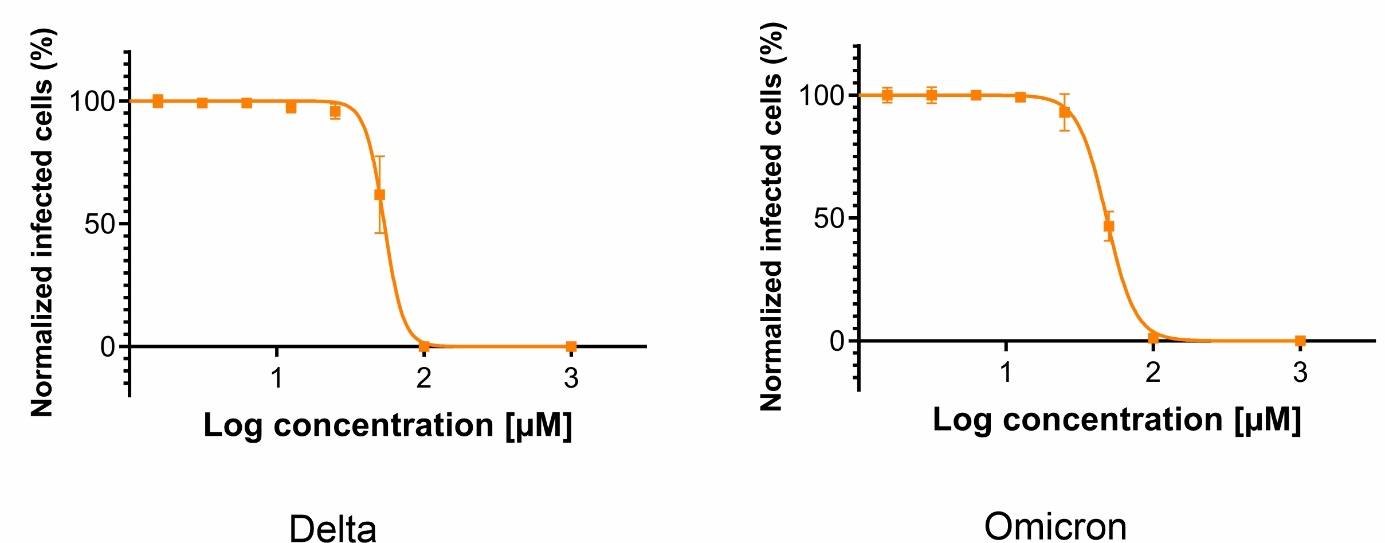
**

**Figure S7:** Immunofluorescence assay for APC against the delta and omicron strains. The orange curve indicates the inhibitory effects on SARS-CoV-2 replication. The concentration–response curves were generated from a representative biological experiment, in which each concentration was measured in three technical replicates. Error bars indicate the standard deviation of the technical replicates.

**Table S1:** The ΔG_b_ and EI values (kcal/mol) of AMS calculated by QMH-L for the binding modes produced by HydroDock calculations for the M2 and EP ion channels.

| **AMS** | **M2**  **(Influenza)** | **EP**  **(SARS-CoV-2)** | **EI**  **(SARS-CoV-2)** |
| --- | --- | --- | --- |
| AMA | -10.0 | -7.3 | -0.66 |
| ART |  | -15.1 | -0.54 |
| MEM |  | -7.5 | -0.58 |
| RIM | -14.3 | -7.2 | -0.56 |
| ETA |  | -12.4 | -0.65 |
| PAC |  | -10.6 | -0.56 |
| APC |  | -6.7 | -0.61 |

**Table S2:** Cytotoxicity, and inhibitory effect of AMS from previously published data

| **AMS** | **EC_50_ [µM]** | **-1.364pEC_50_** | **-1.364pEC_50_/NHA** | **CC_50_ [µM]** | **pCC_50_** | **Ref.** |
| --- | --- | --- | --- | --- | --- | --- |
| Influenza A | | | | | | |
| AMA | 0.78 | -8.33 | -0.76 | >100 |  | [4] |
| RIM | 0.09 | -9.61 | -0.74 | >100 |  | [4] |
| SARS-CoV-2 | | | | | | |
| AMA | 116 | -5.37 | -0.49 | 1411 | 2.85 | [5] |
| ART | 0.2 | -9.14 | -0.33 | >60 | 4.22 | [6] |
| MEM | 80 | -5.59 | -0.43 | 611 | 3.21 | [5] |
| RIM | 36 | -6.06 | -0.47 | 621 | 3.20 | [5] |

**Table S3:** Cytotoxicity, inhibitory effect, and safety index of AMS measured in the present study

| **AMS** | **CC_50_ [µM]** | **EC_50_ [µM]** | | | | **SI (CC_50_/EC_50_)** | | | |
| --- | --- | --- | --- | --- | --- | --- | --- | --- | --- |
|  |  | **Alpha**  **(B.1.1.7)** | **Delta**  **(B.1.617)** | **Omicron**  **(B.1.1.529)** | **Wuhan (P.2)** | **Alpha**  **(B.1.1.7)** | **Delta**  **(B.1.617)** | **Omicron**  **(B.1.1.529)** | **Wuhan (P.2)** |
| 4 AMS drugs | | | | | | | | | |
| AMA | >800 | 31.66 | (415.10) | (414.00) | n.d. | >25 | 1.93 | 1.93 | n.d. |
| ART | 42 | 9.66 | 9.24 | 46.16 | 13.55 | 4.35 | 4.55 | 0.91 | 3.10 |
| MEM | 355 | 159.60 | (321.80) | 318.50 | n.d. | 2.22 | 1.10 | 1.11 | n.d. |
| RIM | 257 | 41.01 | 369.00 | (118.40) | n.d. | 6.27 | 0.70 | 2.17 | n.d. |
| Elongation of the AMA scaffold | | | | | | | | | |
| ETA | 33 | (154.20) | 10.13 | 37.03 | (175.50) | 0.21 | 3.26 | 0.89 | 0.19 |
| PAC | 87 | 40.92 | 14.91 | 7.78 | 4.83 | 2.13 | 5.84 | 11.18 | 18.01 |
| Simplification of the scaffold | | | | | | | | | |
| APC | 229 | 81.13 | 8.32 | 49.42 | 8.16 | 2.82 | 27.52 | 4.63 | 28.06 |

n.d.= not determined

Values in brackets have low statistical reliability.

**Table S4:** Immunofluorescence assay measurement for APC

| **IC_50_ [µM]** | | | |
| --- | --- | --- | --- |
| **Alpha (B.1.1.7)** | **Delta (B.1.617)** | **Omicron (B.1.1.529)** | **Wuhan (P.2)** |
| 66.98 | 53.61 | 47.54 | 8.96 |

**References**

1. Madeira, F.; Madhusoodanan, N.; Lee, J.; Eusebi, A.; Niewielska, A.; Tivey, A.R.N.; Lopez, R.; Butcher, S. The EMBL-EBI Job Dispatcher Sequence Analysis Tools Framework in 2024. *Nucleic Acids Res.* **2024**, *52*, W521–W525, doi:10.1093/nar/gkae241.

2. Lan, J.; Ge, J.; Yu, J.; Shan, S.; Zhou, H.; Fan, S.; Zhang, Q.; Shi, X.; Wang, Q.; Zhang, L.; et al. Structure of the SARS-CoV-2 Spike Receptor-Binding Domain Bound to the ACE2 Receptor. *Nature* **2020**, *581*, 215–220, doi:10.1038/s41586-020-2180-5.

3. Zsidó, B.Z.; Börzsei, R.; Szél, V.; Hetényi, C. Determination of Ligand Binding Modes in Hydrated Viral Ion Channels to Foster Drug Design and Repositioning. *J. Chem. Inf. Model.* **2021**, *61*, 4011–4022, doi:10.1021/acs.jcim.1c00488.

4. Drakopoulos, A.; Tzitzoglaki, C.; McGuire, K.; Hoffmann, A.; Konstantinidi, A.; Kolokouris, D.; Ma, C.; Freudenberger, K.; Hutterer, J.; Gauglitz, G.; et al. Unraveling the Binding, Proton Blockage, and Inhibition of Influenza M2 WT and S31N by Rimantadine Variants. *ACS Med. Chem. Lett.* **2018**, *9*, 198–203, doi:10.1021/acsmedchemlett.7b00458.

5. Zhou, Y.; Gammeltoft, K.A.; Galli, A.; Offersgaard, A.; Fahnøe, U.; Ramirez, S.; Bukh, J.; Gottwein, J.M. Efficacy of Ion-Channel Inhibitors Amantadine, Memantine and Rimantadine for the Treatment of SARS-CoV-2 In Vitro. *Viruses* **2021**, *13*, 2082, doi:10.3390/v13102082.

6. Ghosh, A.K.; Miller, H.; Knox, K.; Kundu, M.; Henrickson, K.J.; Arav-Boger, R. Inhibition of Human Coronaviruses by Antimalarial Peroxides. *ACS Infect. Dis.* **2021**, *7*, 1985–1995, doi:10.1021/acsinfecdis.1c00053.
